# The neural correlates of perceived energy levels in older adults with late-life depression

**DOI:** 10.1007/s11682-018-9940-y

**Published:** 2018-08-29

**Authors:** Charlene L. M. Lam, Ho-Ling Liu, Chih-Mao Huang, Yau-Yau Wai, Shwu-Hua Lee, Jenny Yiend, Chemin Lin, Tatia M. C. Lee

**Affiliations:** 1 State Key Laboratory of Brain and Cognitive Sciences, The University of Hong Kong, Pokfulam, Hong Kong; 2 Laboratory of Neuropsychology, The University of Hong Kong, Pokfulam, Hong Kong; 3 Institute of Clinical Neuropsychology, The University of Hong Kong, Pokfulam, Hong Kong; 4 Department of Imaging Physics, University of Texas MD Anderson Cancer Center, Houston, TX USA; 5 College of Biological Science and Technology, National Chiao Tung University, Hsinchu, Taiwan; 6 Department of Medical Imaging and Intervention, Chang Gung Memorial Hospital, Taoyuan, Taiwan; 7 Department of Psychiatry, Linkou Chang Gung Memorial Hospital, Taoyuan, Taiwan; 8 College of Medicine, Chang Gung University, Taoyuan, Taiwan; 9 Institute of Psychiatry, Psychology and Neuroscience, King’s College London, London, UK; 10 Department of Psychiatry, Chang Gung Memorial Hospital, Keelung City, Taiwan

**Keywords:** Late-life depression, Corticospinal tract, Processing speed, White matter hyperintensities, White matter

## Abstract

Late-life depression is common among older adults. Although white-matter abnormality is highly implicated, the extent to which the corticospinal tract is associated with the pathophysiology of late-life depression is unclear. The current study aims to investigate the white-matter structural integrity of the corticospinal tract and determine its cognitive and functional correlates in older adults with late-life depression. Twenty-eight older adults with clinical depression and 23 healthy age-matched older adults participated in the study. The white matter volume and the white matter hyperintensities (WMHs) of the corticospinal tract, as well as the global WMHs, were measured. Psychomotor processing speed, severity of depression, perceived levels of energy and physical functioning were measured to examine the relationships among the correlates in the depressed participants. The right corticospinal tract volume was significantly higher in depressed older adults relative to healthy controls. Moreover, the right corticospinal tract volume was significantly associated with the overall severity of depression and accounted for 17% of its variance. It further attenuated the relationship between the severity of depression and perceived levels of energy. Our findings suggested that higher volume in the right corticospinal tract is implicated in LLD and may relate to lower perceived levels of energy experienced by older adults with depression.

Depression occurring in late-life (LLD) is common, with an estimated prevalence of 7.2% for clinical major depression and 17.1% for depressive disorders (Luppa et al. 2012). It is an area of intense clinical research because of its high prevalence in the older population, as well as the aging populations that many countries are now confronting. The course of LLD is often chronic and devastating to quality of life. More than 50% of the older adults with depression continue to experience depressive symptoms two years after the diagnosis (Blazer 2003), and 35% of the cases are resistant to antidepressant medications (Gottfries 2001). Given the high social and economic costs associated with LLD, understanding the neural mechanism of LLD is essential for providing insights that could lead to the development of more effective diagnosis and intervention.

One distinct feature of late-life depression is psychomotor disturbance and loss of energy (Parker et al. 2001). The neural mechanism of these behavioral symptoms of depression is yet to be elucidated in the literature. One prominent model that explains the psychomotor disturbance and poor executive control is the dysregulated frontal-striatal pathway (Alexopoulos 2005; Tadayonnejad and Ajilore 2014). The frontal-striatal pathway connects the frontal regions to basal ganglia and the thalamus. A number of studies have reported dysfunction of this frontostriatal pathway in LLD. Ballmaier et al. (2004) reported low volumes in the anterior cingulate and gyrus rectus in elderly patients with depression. Volumetric reduction in the striatum and cortical nodes of corticostriatal circuits such as dorsolateral prefrontal cortex, orbitofrontal cortex, and anterior cingulate cortex were also reported in LLD (Furman et al. 2011; Heller et al. 2009; Marchand and Yurgelun-Todd 2010).

Apart from the dysregulated frontostriatal pathway, the vascular depression hypothesis has also been proposed to account for the psychomotor disturbance in LLD. This model posits that cerebrovascular changes in the white matter (WM) are crucial in the pathogenesis of LLD (Alexopoulos 2005; Alexopoulos et al. 1997). Compared to non-vascular depression, patients with vascular depression had greater executive function impairment, psychomotor retardation, poor insight and disability (Alexopoulos et al. 1997). The vascular depression hypothesis has been supported by several neuroimaging studies that reported higher white matter hyperintensities (WMHs) in the prefrontal regions in patients with LLD (Firbank 2004; Tham et al. 2011). Herrmann and colleagues (2007) conducted a meta-analytic study on WM changes in LLD and reported that WMHs are more frequent and intense among patients with LLD. Furthermore, there is evidence that higher WMHs are associated with a decline in executive skills, attention, and processing speed (Au et al. 2006; Gunning-Dixon and Raz 2000; Van Den Heuvel et al. 2006). However, there is a dearth of research examining the WMHs in the corticospinal tract of patients with LLD (Fig. 1).

**Fig. 1:**
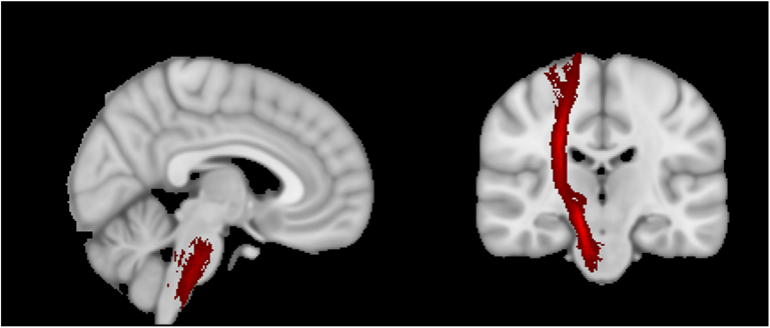
Location of the right corticospinal tract (Left: Sagittal view; Right: Coronal view)

Emerging evidence has shown support for the involvement of brainstem in major depressive disorder (Sacchet et al. 2014a, b; Song et al. 2014). The brainstem is the main region that innervates neurotransmitter release to both the fronto-limbic system and the hypothalamic-pituitary-adrenal (HPA) axis, which are both implicated in the theories of depression (Geerlings and Gerritsen 2017; Gotlib et al. 2008; Pariante and Lightman 2008). Using diffusion tensor tractography, Song et al. (2014) observed a decreased fractional anistropy (FA) in the solitary tract of the brainstem tract in depressed patients relative to healthy controls. In a longitudinal imaging study using whole-brain voxel-wise approach, Soriano-Mas et al. (2011) reported a volumetric increase in the white matter of the upper brainstem tegmentum and a volumetric reduction in the periventricular white matter in a group of melancholic patients. More recently, Sacchet et al. (2014a, b) documented a higher FA in the bilateral corticospinal tract of patients with depression. The nature of the WM abnormality in the CST remains indeterminate. It was postulated that the altered WM integrity may reflect an abnormal connectivity between subcortical and brainstem structures and cortical regions. Specifically, the corticospinal tract is a major white matter tract running from the spinal cord through the brainstem to the primary motor cortex. It is important for voluntary control of both the upper and lower limbs, as well as modifying sensory impulses to regulate ascending information (Blumenfeld 2010). Lesions of the corticospinal tract may interfere with motor processes, given its extensive connection from the brainstem to the gray matter in the cortical motor regions.

Existing literature is scarce on the structural integrity of the corticospinal tract in LLD. The present study was undertaken to explore the WM macrostructural integrity in the corticospinal tract in LLD. This study extended the previous studies by using analyses based on regions of interest (ROIs) to examine the findings on the abnormality of corticospinal tracts in LLD and explore its cognitive and functional correlates. Since WMHs are prevalent in the elderly and are implicated in LLD (Au et al. 2006; Herrmann et al. 2008; Smagula and Aizenstein 2016; Tham et al. 2011), we compared both the WMHs and WM volume of the corticospinal tracts between the depressed older adults and their age-matched healthy controls. Moreover, we measured participants’ psychomotor processing speed, self-perceived physical functioning, and perceived levels of energy as behavioral correlates. In light of the existing literature, we hypothesized that higher WMHs and reduced WM volume would be observed in the corticospinal tract of patients with LLD in comparison to the control group. We also predicted that WM volume and WMHs of the corticospinal tract would correlate with the psychomotor processing speed and physical functioning among the depressed older adults.

## Methods

The present study was part of a larger fMRI study with the aim to examine the role of different cognitive and neuroimaging factors in depressed older adults (Wong et al. 2016). The current study was approved by the Institutional Review Board in the Chang Gung Memorial Hospital. All participants signed an informed consent prior to their participation. All procedures performed were in accordance with the ethical standards of the institutional and/or national research committee and with the 1964 Helsinki declaration and its later amendments or comparable ethical standards.

### Participants

Twenty-eight right-handed older adults and 23 age-matched, healthy adults (the control group) were included in this study. All participants had normal or corrected-to-normal vision. Participants who scored lower than 24 in the Mini-Mental State Examination or who had a history of neurological diseases were excluded to avoid the potential confounding effects of dementia or other neurological illnesses. The clinical population was recruited from the psychiatric outpatient clinic of a local hospital. Healthy older adults were recruited from local communities and same exclusion criteria were adopted both for the clinical and the control groups. Two geriatric psychiatrists diagnosed major depressive disorder according to the criteria listed in the *Diagnostic and Statistical Manual of Mental Disorders Fifth Edition* (DSM-5; APA 2013). Patients with comorbidity of other psychiatric illnesses were excluded. All patients experienced their first major depressive episodes after age 60. Antidepressants were maintained at the time of the scans for ethical reasons; each participant’s medication level was kept unchanged for at least two weeks before his or her scanning session. The demographic information of our participants is shown in Table 1.

**Table 1:** Demographic and clinical characteristics of patients with late-life depression and healthy controls

	Depressed (*n* = 28)	Healthy (*n* = 23)	*t*-value
	Mean (SD)	Mean (SD)	
Age (year)	68.25 (4.97)	67.13 (4.79)	0.81
Gender (male/female)	9/19	9/14	
Age of onset (year)	64.18 (3.81)	–	
Number of episodes	1.36 (0.56)	–	
MMSE	27.00 (2.61)	27.87 (1.63)	1.39
GDS	7.15 (3.65)	3.35 (1.92)	4.48*
TIV (ml)	1338.23 (129.62)	1314.81 (125.01)	0.65

*MMSE* Mini Mental State Examination, *GDS* Geriatric Depression Scale, *TIV* Total intracranial volume

* *p* < .05

### Psychological measure

The Geriatric Depression Scale – Short Form (GDS; Chiu et al. 1994) is a 15-item self-report assessment used to identify the symptoms and severity of depression in older adults. Some examples of the questions are “Do you often feel helpless?” and “Do you feel full of energy?” Participants were required to answer “yes” or “no” to the questions. Higher scores denote more depressive symptoms and reflect higher severity of depression. This scale has been previously validated in the Chinese population (Wong et al. 2002).

### Cognitive measure

The Digit-Symbol Coding subtest of the Chinese version of the Wechsler Adults Intelligence Scale - III (Chen and Chen 2002) was used to estimate the psychomotor processing speed. In this task, participants were required to copy a symbol that was paired with a number under timed conditions. Higher scores represent better performance.

### Perceived physical functioning

Short-Form Health Survey-36 (Lam et al. 1998) is a 36-item self-reported health survey covering various health-related issues, including physical functioning, social functioning, role limitations (physical and emotional); mental health; perceived levels of energy; bodily pain; and general health perceptions. For this study, we focused on the scales for physical functioning and perceived levels of energy. Sample questions regarding physical functioning include “Does your health limit your ability to climb flights of stairs?” and “Does your health limit your ability to lift or carry groceries?” Sample questions regarding the perceived levels of energy include “Did you feel tired?” and “Did you feel worn out?”. Each scale sums to 100. Higher scores indicate lower levels of disabilities.

### MRI data acquisition and preprocessing

The T1-weighted structural images were acquired using a 3 Tesla MR 750 scanner (GE Healthcare Systems, Waukesha, WI, USA) with an 8-channel head coil. An inversion-recovery prepared fast 3D spoiled gradient-echo sequence with the following parameters was employed to collect the structural imaging data: whole brain, matrix size = 256 × 256, repetition time = 8 ms, echo time = 3 ms, inversion time (TI) = 450 ms, 160 slices, flip angle = 12 degrees, field of view = 250 × 250 mm^2^, and voxel size = 0.98 × 0.98 × 1 mm^3^. All preprocessing was performed with the VBM 8 toolbox (http://dbm.neuro.unijena.de/vbm/download) in Statistical Parametric Mapping 12 (Wellcome Department of Imaging Neuroscience, London, UK; http://fil.ion.uck.ac.uk/spm) using Matlab (Mathworks Inc., Natick, MA, USA).

First, we used the standard unified segmentation module in Statistical Parametric Mapping 12 (Ashburner and Friston 2005) to segment the MR images into gray matter, WM, and cerebrospinal fluid. Second, we employed the DARTEL procedure (Ashburner 2007) to determine the nonlinear deformations for warping all WM or gray-matter images in order to register individuals to the standard space. After an initial affine registration of the WM DARTEL templates to the tissues’ probability maps in the Montreal Neurological Institute (MNI) space, a nonlinear warping of WM images was performed to normalize the DARTEL WM templates in MNI space. The MR images were then modulated to preserve the relative volumes of WM following the spatial-normalization procedure. Finally, we smoothed the images using an 8-mm full-width-at-half-maximum Gaussian kernel. After this spatial preprocessing, the smoothed, modulated, and normalized WM data were used for statistical analyses.

For subsequent ROI analyses, we anatomically defined ROIs using the JHU WM tractography atlas (http://cmrm.med.jhmi.edu/). We extracted participants’ WM volume within the corticospinal masks and performed regression analyses to examine their relationships with the cognitive and physical functioning measures.

### WMHs segmentation and localization

A fully automated method was employed for quantification and localization of WMHs (Wu et al. 2006). We obtained the WMH masks from the coregistered T2-weighted FLAIR image for each participant. We applied a fuzzy-connected algorithm to automate the segmentation of WMHs, which involved the following four steps: (1) automatic identification of WMH seeds based on the intensity histogram of the FLAIR image, (2) segmentation of WMH clusters using a fuzzy connected algorithm based on their adjacency and affinity, (3) iterative update of the set of seeds, and (4) combination of WMH clusters into final WMH segmentation. The fully automated WMH segmentation was carried out in C++ and Insight Segmentation and Registration Toolkit (ITK; Yoo 2004). The WMH segmentation was then normalized to MNI space (2 mm isotropic) using the deformation field generated in SPM12. Demon-Based image registration was employed to automate the anatomic localization of the WMHs using the JHU WM atlas. WMHs volume (mm^3^) in the bilateral corticospinal tracts, and the whole brain were obtained for group comparison in the current study.

### Medication load

To control the effect of medication in our study, we computed the medication load for each participant according to Antidepressant Treatment History Form ATHF-modified (Sackeim 2001). Each antidepressant was rated on a 4-point scale based on the type of antidepressants and the course of treatment. The concomitant use of hypnotics was also coded from 0 to 1. The sum of antidepressant and hypnotic loads were used as the medication load in our study.

### Statistical analysis

First, we calculated F-statistics to compare the group differences in the WM volume of the corticospinal tracts, the whole brain WMHs volume, psychomotor processing speed, physical functioning and perceived levels of energy. We controlled for participants’ age and total intracranial volume when comparing the group differences in the WM volume and the WMHs volume. We controlled for participants’ age when comparing the group differences in their psychomotor processing speed, physical functioning and perceived levels of energy.

Second, we performed a Chi-square test of Independence to examine the association between groups (depressed patients vs. healthy controls) and the presence of WMHs volume because the distribution of the WMHs volume for the bilateral corticospinal tract were positively skewed and 64% of the cases had zero WMHs volume.

Third, we examined the association of the WM volume in the corticospinal tract, and the whole brain WMHs volume using Pearson’s correlation and step-wise multiple linear regression. We controlled for age, total intracranial volume and the medication load in these analyses.

To explore the direct and indirect effects of depression severity on perceived levels of energy, we performed ordinary least square regression to estimate coefficients in the model. We submitted the WM volume of the right corticospinal tract to the model to test its impact on the association between depression severity and perceived energy levels. Bootstrapping using 5000 samples was used to generate a confidence interval for the direct and indirect effects. This analysis was conducted using PROCESS (model 4) for SPSS (Hayes 2013). In this analysis, we controlled for participants’ age, total intracranial volume and their medication load.

All statistical analyses were performed in SPSS 20.0 (SPSS Inc., Chicago, IL, USA), and the statistical significance was set at *p* < .05.

## Results

### Corticospinal tract WM volume, WMHs volume, processing speed, and perceived physical functioning

Comparing to the healthy controls, depressed older adults had a significantly higher WM volume in the right corticospinal tract (*F*_1,47_ = 4.47, *p* = .04). The difference in WM volume in the left corticospinal tract approached statistical significance (*F*_1,47_ = 3.33, *p* = .07). There were no significant group differences in their total intracranial volume (*t* (49) *= .65*, *p* > .05).

Depressed older adults did not have higher whole brain WMHs volume comparing to the healthy controls (*F*_1,39_ = 0.26, *p* > .05).With respect to the WMHs volume in the bilateral corticospinal tract, we did not observe any WMHs in 68.2% of the patients and 52.4% of the healthy participants. The median of the WMHs volumes of the bilateral corticospinal tracts were zero for both groups. A chi-square test of independence suggested that there were no group difference in the WMHs volume of both left and right corticospinal tracts (Left CST: *χ*^2^ (1) = .19, *p* = .67; Right CST: *χ*^2^ (1) = 1.12, *p* = .29).

The depressed older participants had a significantly slower psychomotor processing speed (*F*_1,48_ = 9.45, *p* = .003) than the healthy controls. They also reported significantly lower perceived levels of energy than the healthy adults (*F*_1,48_ = 16.33, *p* = .0002). However, there was no significant difference in their physical functioning (*F*_1,48_ = 2.97, *p* > .05) (Table 2).

**Table 2:** Group differences in corticospinal tract white matter volume, white matter hyperintensities, psychomotor processing speed, and perceived health status

	Depressed (*n* = 28) Mean (SD)	Healthy controls (*n* = 23) Mean (SD)	*F*	*p*-value
Corticospinal tract white matter volume ^*a*^				
Left	.7523 (.0612)	.7247 (.0423)	3.33	.074
Right	.7484 (.0597)	.7171 (.0444)	4.47	.040
WMHs volume (whole brain) (mm^3^) ^*a*^	5258.64 (5515.16)	4261.81 (5348.88)	0.26	0.61
Psychomotor processing speed ^*b*^				
Digit-Symbol Coding	18.75 (7.47)	25.74 (7.94)	9.45	.003
Perceived health status ^*b*^				
Physical functioning	72.50 (26.54)	85.00 (23.01)	2.97	.092
Perceived energy level	46.35 (18.90)	70.43 (21.42)	16.33	.0002

^a^ Controlled for age and total intracranial volume; ^*b*^ controlled for age

### Correlates of corticospinal tract WM volume and whole brain WMHs volume with cognitive and physical functioning measures

Table 3 describes the Pearson’s correlates of the corticospinal WM volume. There were no significant correlations between WM volume in the corticospinal tracts and psychomotor processing speed among the depressed older adults in this study (*ps* > .05). However, the severity of depression correlated significantly with the left corticospinal WM volume (*r* = .48, *p* = .03), right corticospinal WM volume (*r* = .49, *p* = .03), and perceived levels of energy (*r* = −.53, *p* = .02). The whole brain total WMHs volume was negatively associated with psychomotor processing speed (*r* = −.56, *p* = .02).

**Table 3:** Pearson’s Correlation of corticospinal tract white matter volume, white matter hyperintensities, processing speed and perceived health status

	GDS	Left corticospinal tract WMV	Right corticospinal tract WMV	Whole brain total WMHs	Digit-Symbol Coding	Physical functioning	Perceived energy level
GDS	–						
Left corticospinal WMV	.48*	–					
Right corticospinal WMV	.49*	.97**	–				
Whole brain total WMHs	.03	−.06	−.06	–			
Digit-Symbol Coding	.03	.28	.20	−.56*	–		
Physical functioning	.20	.22	.20	−.42	.32	–	
Perceived energy level	−.53*	−.07	−.03	−.32	.23	.08	–

All measures controlled for age, total intracranial volume and medication load; GDS: Geriatric Depression Scale; WMV: white matter volume; WMHs: white matter hyperintensities

* *p* < .05, ** *p* < .01

### WM volume of the corticospinal tract, severity of depression, and perceived levels of energy

The WM volume in the right corticospinal tract was significantly associated with the severity of depression (*β* = .46, *t* (20) = 2.33, *p =* .03). The inclusion of right corticospinal tract volume explained 17% of the variance in the severity of depression (Δ*R*^2^ = .17, *F*_1,20_ = 5.45, *p* = .03).

**Fig. 2:**
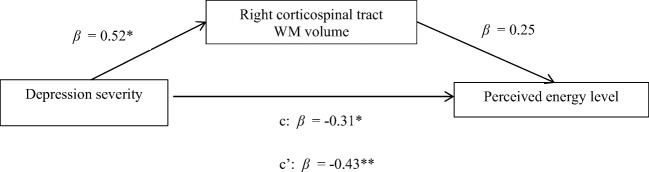
The effect of right corticospinal tract volume on the relationship between depression severity and perceived energy level. Standardized regression coefficient of each path is reported. * *p* < .05; ** *p* < .01. c represents the regression coefficient of depression severity on perceived level before including right corticospinal tract WM volume into the equation; c’ the value after including right corticospinal tract WM volume

Furthermore, there was a significant indirect effect of depression severity on perceived levels of energy through the right corticospinal tract WM volume (*β* = 0.13, SE = 0.08, 95% CI [0.0063, 0.3120]). Standardized regression coefficients of each path were reported in Fig. 2. Depression severity was positively correlated with the right corticospinal tract WM volume (*β* = 0.52, *p* = 0.02) and a positive trend was observed between the right corticospinal tract volume and participants’ perceived levels of energy (*β =* 0.25, *p* = .08). Before including the right corticospinal tract WM volume in the model, the total effect of depression severity on perceived energy levels was −0.31 (SE = 0.14, 95% CI [−0.609, −0.012]). After including the right corticospinal tract WM volume in the model, the effect of severity of depression on participants’ perceived level of energy level was attenuated (*β* = −0.43, SE = .15*,* 95% CI [−0.739, −0.114].

## Discussion

In this study, we examined the macrostructural integrity of WM in the corticospinal tract of older adults with clinical depression and explored its cognitive and functional correlates. Our findings revealed that older adults with LLD had significantly higher WM volume in the right corticospinal tract than the age-matched healthy controls. Furthermore, the WM volume of the right corticospinal tract attenuated the association between the severity of depression and perceived levels of energy.

To our best knowledge, this is the first study to demonstrate a volumetric alteration in the right corticospinal tract of older adults with LLD. In our data acquisition, WMHs appear hypodense in T1. Thus, any mis-segmentation would result in WMHs being mis-classified as gray matter, not WM. We are therefore confident that the finding in the WM volume of the right corticospinal tract cannot be influenced by WMH. Our findings concur with previous reports on the implication of corticospinal tract in major depressive disorder (Sacchet et al. 2014a, b). Although the aetiology behind the WM volumetric expansion in the corticospinal tract remains unclear, this aberration in the WM may be a harbinger of WMHs in this particular region. In a longitudinal study that followed a group of depressed patients over 3.5 years, De Groot et al. (2013) observed early microstructural changes in the normal appearing white matter (NAWM) regions which were later converted to WMHs. Their findings provide crucial insight into the pathophysiology of WMHs: the development of WMHs is likely a gradual process and changes in WM may have already been present before they become visually appreciable or computationally identifiable as WMHs on MRI scans. The volumetric increase of WM in the right corticospinal tract observed in our depressed patients may reflect this early stage of development of WMHs; however this assertion can only be substantiated with a longitudinal research design and analysis of brain images with different methodologies (e.g., with diffusion tensor imaging).

The implication of the corticospinal tract and to a larger extent the brainstem in LLD has been largely overlooked by previous structural imaging studies. In addition to studies that observed a macrostructural change in the WM of the brainstem (Lee et al. 2011; Soriano-Mas et al. 2011), numerous reports have also documented the microstructural WM alteration in this region in depressed patients. For instance, Abe et al. (2010) observed a higher mean diffusivity in the pons of the brainstem while Song et al. (2014) reported a higher fractional anisotropy in the right solitary tract of the brainstem. Increased connectivity from the amygdala to the hippocampus, cerebellum and the brainstem has also been reported (Arnold et al. 2012). These studies have offered solid support to the implication of brainstem in LLD.

In our study, we did not observe a higher global burden of WMHs in the depressed older adults relative to the healthy controls. This was somewhat unexpected, given higher WMHs has been documented in LLD in many previous reports (Herrmann et al. 2008; Smagula and Aizenstein 2016). We believe the null finding may be related to factors such as unmatched cerebrovascular risk for our participants and the heterogeneity of depression in our sample (e.g., differences in onset, duration, and severity of depressed episodes). Nonetheless, we observed a significant association between the global WMHs and psychomotor processing speed. This finding was consistent with the general finding in the literature (Debette and Markus 2010; Van Den Heuvel et al. 2006), supporting the implication of WMHs in cognitive functions such as processing speed. The null association between corticospinal tract WM volume and processing speed in our study supported the notion that psychomotor processing speed correlates with the integrity of the WM in the whole brain (Magistro et al. 2015; Penke et al. 2010). Impairment in processing speed is an area of high concern, as processing speed is a core deficit in the cognitive profile of LLD (Sheline et al. 2006). Previous studies suggested that depression may exert an additional effect on the decline of processing speed in depressed older adults apart from old age (Mettenburg et al. 2012). Older adults with depression often exhibit persistent and substantial cognitive impairments, even after their mood symptoms have improved (Bhalla et al. 2006; Köhler et al. 2010; Lee et al. 2007). In our study, depressed older adults had a reduced processing speed compared to the healthy controls. Given the association between WMHs and processing speed, physicians or other mental health workers should consider referring patients with LLD for routine screening for WMHs-related illnesses such as stroke and dementia.

Our findings highlight the attenuating role that the right corticospinal tract volume may play in the relationship between the severity of depression and perceived levels of energy. Although no causality can be inferred, participants’ perceived low energy levels may relate to the volumetric alteration in the right corticospinal tract. This is a novel finding in the literature and offers a neural basis to account for the behavioral symptom commonly reported by depressed older adults. Previous studies suggested that fatigue can be a mental perception as well as a physical manifestation, and that it involves a contribution from the cortical motor and somatosensory areas (Gruet et al. 2013). We speculate that the alteration of the corticospinal tract volume may interrupt effective communication between the cortical motor areas and the limbs, contributing to the low levels of energy or fatigue that depressed patients experienced. Moreover, a recent DTI study on the WM integrity of the corticospinal tract across different age groups revealed that the fiber number of corticospinal tract connecting to the secondary motor area (M2) decreased significantly in the 70s age group (Jang and Seo 2015). The depressed patients in our study were relatively young and their mean ages were below 70 years. Future studies may consider recruiting older participants with LLD and examining the interaction effect of age and severity of depression on the WM integrity of the corticospinal tract.

### Limitations

This study has a few limitations. First, the medications’ effects on the cognitive and functional measures were unknown. For ethical reasons, we did not exclude participants who were on medication. However, it was almost impossible to control for many types of antidepressant medications in our statistical analysis. Researchers conducting further studies may include treatment-naïve participants to achieve a more homogenous sample. Second, we used an atlas-defined ROI to locate the positions of the corticospinal tracts and to compute the WM volumes. Researchers have argued that whole-brain voxel-wise morphometry and comparison can eliminate the operator’s bias. Because we were interested in an a priori region, we believed that we were justified in using the ROI method to investigate the cognitive and functional correlates of corticospinal tracts in this study. Third, other tracts are commonly implicated in depression, including the uncinate fasciculus and the superior longitudinal fasciculus; those tracts may interact with the cognitive and functional correlates. Researchers, in further studies, should consider using a multimodal neuroimaging approach to identify and compare different ROIs to fully comprehend the structural and functional connectivity of the frontal-subcortical network and how that affects depression in older adults. Lastly, the sample size of the clinical population in our study is small. Despite the small sample, it represents a more homogenous group of depressed older adults who are free of neurological or other psychiatric illnesses. Given the high comorbidity of physical and psychiatric illnesses in older adults, the exclusion criteria in our study inevitably limit the generalizability of our findings. Future studies should include more participants and relax the exclusion criteria to enhance the generalizability of the findings of this study.

## Conclusions

In this study, we demonstrated that aberrance in the right corticospinal tract volume was associated with the severity of depression in a group of clinically depressed older adults. The corticospinal tract WM volume attenuated the relationship between depression severity and perceived levels of energy in this clinical population. Our study contributes to our understanding of the structural abnormality of corticospinal tracts in LLD, and our findings suggest that researchers need a more thorough understanding of LLD’s WM pathology and the effects of corticospinal tract WM volume in order to better understand the neural mechanism of the illness.
